# Ecological Diversity of Bacterial Rhizomicrobiome Core during the Growth of Selected Wheat Cultivars

**DOI:** 10.3390/biology12081067

**Published:** 2023-07-30

**Authors:** Agnieszka Kuźniar, Kinga Włodarczyk, Sara Jurczyk, Ryszard Maciejewski, Agnieszka Wolińska

**Affiliations:** 1 Department of Biology and Biotechnology of Microorganisms, The John Paul II Catholic University of Lublin, Konstantynów St. 1 I, 20-708 Lublin, Poland; kinga.wlodarczyk@kul.pl (K.W.); agnieszka.wolinska@kul.pl (A.W.); 2 Department of Artificial Intelligence, The John Paul II Catholic University of Lublin, Konstantynów St. 1 H, 20-708 Lublin, Poland; sara.jurczyk@kul.pl; 3 Department of Human Anatomy, Medical University of Lublin, Jaczewskiego 4 St., 20-950 Lublin, Poland; ryszard.maciejewski@umlub.pl; 4 Institute of Health Sciences, The John Paul II Catholic University of Lublin, Konstantynów St. 1 H, 20-708 Lublin, Poland

**Keywords:** rhizomicrobiome core, wheat, rhizospheric soil, bioinformatic analysis

## Abstract

**Simple Summary:**

The rhizosphere is the narrow region of soil that is directly influenced by root secretions and their associated microorganisms, named as the root microbiome. The rhizosphere is known as the niche most rich in microorganisms. From a microbiological point of view, the identification of these microorganisms and the discovery of the functions they perform by living, for example, in symbiosis with plants, is of interest. Consequently, this study aims to analyze the diversity of soil bacteria inhabiting the rhizospheric zone of wheat (*Triticum aestivum* L.), represented by four wheat cultivars (Tytanika, Nordcap, Hondia, Rotax), employing three methods of microbiome determination: (1) membership, (2) composition, and (3) functionality. Wheat cultivation in Poland is cropped on about 20% of all arable land. The studied cultivars are characterized by good yielding parameters and are highly popular for growing in Poland. A technique independent of cultivation (NGS—Next Generation Sequencing) was applied for microbial biodiversity determination, whereas for prediction of their ecological functionality, the FAPROTAX database was utilized. By performing lab analyses, we evidenced that molecular identification of the core rhizomicrobiome is particularly important for understanding the general principles regarding the selection of microorganisms around living roots and for harnessing the power of the microbiome in agricultural practice.

**Abstract:**

One of the latest ecological concepts is the occurrence of a biased rhizosphere of microorganisms recruited mostly through interactions among various components of the rhizosphere, including plant roots and the bulk soil microbiome. We compared the diverse attributes of the core microbiome of wheat rhizosphere communities with wheat (W) and legume (L) forecrops determined by three different methods in this study (membership, composition, and functionality). The conclusions of the three methods of microbiome core definition suggest the presence of generalists, i.e., some representative microorganisms from Proteobacteria, Actinobacteria, Hypomicrobiaceae, Bradyrhizobiaceae, *Sphingomonas* sp., in the wheat rhizomicrobiome. The relative abundance of the core microbiome accounted for 0.1976% (W) and 0.334% (L)—membership method and 6.425% (W) and 4.253% (L)—composition method. Additionally, bacteria of the specialist group, such as *Rhodoplanes* sp., are functionally important in the rhizomicrobiome core. This small community is strongly connected with other microbes and is essential for maintenance of the sustainability of certain metabolic pathways.

## 1. Introduction

Soil is a complex of numerous organic and inorganic materials, including minerals, water, gas, and organic matter, and is thus considered one of the most complicated natural systems on Earth [1]. This environment provides home to a wide range of microorganisms, including nematodes, bacteria, fungi, yeasts, archaeons, and algae, as well as insects, various invertebrates, and plants [2,3,4]. Bacteria are the most numerous group. The number of bacterial phylotypes per gram of soil varies between 10^−2^ and 10^−6^, and their greatest abundance and diversity is detected in the upper layers of the soil, but they decline with depth [2,5]. Of note, the chemical composition, moisture, acidity (pH), oxygen, toxic compounds, and structure of soil influence the prevalence of microorganisms [3,6]. Furthermore, the number of microorganisms and their biodiversity is considered a good indicator of soil quality [6,7,8].

All soil organisms are an integral part of the soil and form the basis for its proper functioning [6]. Microorganisms play an essential role in numerous processes in the soil. They are responsible for the bulk of soil enzymatic reactions, use their biomass to store energy and nutrients, and participate in biological, microbial, chemical, and biochemical processes [9,10]. The enormous metabolic diversity of soil microorganisms means that they participate in the cycling of all the major elements, including carbon, nitrogen, and phosphorus, i.e., compounds necessary for the production of amino acids, proteins, and nucleic acids [11]. Soil microorganisms are involved in biogeochemical cycles and regulate such soil processes as mineralization and decomposition of organic matter and the associated release of carbon dioxide, methane, and nitrous oxide into the atmosphere [9,10]. In turn, the microbes present in soil aggregates influence the distribution of oxygen in the soil. In this way, they create habitats for anaerobic microorganisms that catalyze denitrification processes or methane production [2]. Furthermore, rhizosphere microorganisms can live in symbiosis with plants. By producing numerous enzymes, biologically active compounds, and antibiotics, they improve plant health and protect plants against pathogens [6,9]. Soil microorganisms shape the soil structure and create favorable conditions for both seed germination and the growth of the root system of cultivated plants [12]. Thanks to their numerous plant-stimulating properties, soil microorganisms are of crucial importance to agriculture. They not only have a positive effect on plant health but also improve soil quality, enabling farmers to cultivate crops in a sustainable and environmentally sound manner [13]. The interaction between the soil environment and its microbiome affects both the functioning and stability of the soil and the microbial community [3,7]. Microbial activity is essential for fertile and good-quality soil [14]. Determination of the structure and, more importantly, the function of the soil microbiome are essential for elucidation of the impact of soil environmental processes on crop growth, especially as this relationship is not yet well understood [15]. For some time now, the term ‘core microbiome’ has been used in the literature to refer both to the microbiome of humans, animals, and plants and to the microbiome of soils, waters, or wastewater [16]. Originally, this concept only referred to the taxonomy of microorganisms, while the development of techniques based on metagenomics and metatranscriptomics allowed the functions of microbial communities to be defined based on the presence of key genes [17,18]. The communities of bacteria, fungi, archaeons, and protists that make up the microbiome interact with the host plant at different compartments [19]. The interaction between plant roots and the rhizosphere is particularly important. It is widely recognized that the rhizosphere microbiome is of great importance for plant growth and health, and deliberate manipulation of the rhizosphere microbiome contributes to improved plant nutrition and increased resistance to pathogens and abiotic stresses. Thus, it allows achievement of more sustainable agricultural production systems [19,20,21]. Numerous studies have shown that the main factors influencing the core microbiome include the soil type, the host plant genotype, and environmental conditions, and the functional microbiota of rhizospheric soil can be inherited both vertically and horizontally [16,20,22,23]. In recent years, the concept of the rhizospheric soil microbial core has attracted considerable interest due to the possibility of exploiting microbiome functions in agroecosystems [17]. Identifying permanent members of the community is possible using next-generation sequencing (NGS) [19]. NGS is a high-throughput sequencing technology used in metagenomic studies of complex microbial communities [23]. This technology facilitates in depth analysis of microbial communities from taxonomic and phylogenetic points of view [24,25,26]. Characterization of the core rhizospheric microbiome should be carried out at the highest possible taxonomic level, as the metabolism and ecology of closely related taxa may differ [27]. Fortunately, the available bioinformatic methods provide new opportunities to identify thousands of microbial taxa at appropriate taxonomic levels [19]. There is still no knowledge of the consortia of microorganisms that make up the core wheat rhizospheric microbiome, which plays a key role in specific cropping systems [28]. The identification of its composition will allow for the development of crop management practices that will have a consistent impact on the microbial community and thus on sustainable agricultural production [20,29]. Recently, the determination of the core microbiome in agroecosystems mainly targeted at maximization of the utilization of microbiome function has received significant attention. In this context, the main goals of this study are (1) to determine the composition of the soil core microbiome in the rhizosphere during the growth of four wheat cultivars (Tytanika, Nordcap, Hondia, Rotax) planted with different forecrops with application of a culture-independent approach and (2) to predict the functions of the microbiome using the FAPROTAX database.

## 2. Materials and Methods

### 2.1. Rhizospheric Soil Sampling and Description of Experimental Fields

The Haplic Podzol (according to the FAO classification—Food and Agriculture Organization of the United Nations) soil material was collected in autumn (October 2019) from experimental fields (51°24′33″ N, 22°03′06″ E) belonging to the Lublin Agricultural Advisory Center (LAAC) in Końskowola, SE Poland. This region is characterized by a moderately continental climate with an average annual temperature of 7.6 °C and rainfall of approx. 600 mm [29].

Four neighboring plots (each c.a. 130 m^2^) planted with four different winter wheat (*Triticum aestivum* L.) varieties: Hondia (1), Nordcap (2), Rotax (3), and Tytanika (4) with wheat (W) and legume (L) forecrop were selected for this study. The selection of the varieties for the study was based on their good yielding parameters and their high popularity in cultivation in Poland. Importantly, the following crops were rotated at the study area: root crops; spring cereals; legumes; winter crops; rapeseed [29]. Pre-sowing autumn fertilization was applied in September as: N—21 kg∙ha^−1^; P_2_O_5_—60 kg∙ha^−1^; K_2_O—84 kg∙ha^−1^ in the form of Yara Mila. In order to protect the plants, pre-sowing herbicide (Complete 560 SC—0.5 L∙ha^−1^) was also applied. Sowing of all species was carried out with a 3 m wide Poznaniak seeder with foot coulters. The seeds were sown at a row spacing of 12 cm. The experiments were in the nature of a field.

The soil material (rhizosphere soil) was sampled from the rhizosphere (adjacent to the plant; careful sampling so that the soil was root-free) of plants at the BBCH 13 wheat growth stage—a stage with three leaves unfolded (the BBCH scale describes the phenological development of wheat). The samples were pooled in individual plastic bags and transferred into a portable refrigerator [29]. The BBCH 13 stage was chosen deliberately, as this growth stadium is known as critical for winter wheat, because wheat plants usually begin wintering after this stage [29].

From each of the four experimental fields, 25 subsamples were taken from the surface layer (0–15 cm) and combined as 1 sample (biological replicate). In particular, 10 × 10 m squares were chosen in each of the four studied plots. Single samples were collected according to the rules and sampling patterns specified in PN-0431 (Polish Norm dedicated to soil sampling for biological experiments).

Rhizosphere soils studied were characterized by pH: 5.20; 6.27; 7.36; and 6.19, and by the following total organic carbon (TOC) content: 1.09%; 0.74%; 0.76%; and 0.60% for Hondia, Nordkap, Rotax, and Tytanika, respectively [28]. The dominant form of nitrogen was N-NO_3_ ranging from 3.07–4.83 mg∙kg^−1^ followed by NH_4_-N in the range of 0.45–1.11 mg∙kg^−1^ [29].

In laboratory conditions, the fresh soil rhizosphere samples were immediately sieved through a 2 mm sieve and stored shortly (no longer than 24 h) at 4 °C in sterile tubes until DNA extraction [29].

### 2.2. DNA Isolation Procedure

For the extraction of DNA from the rhizospheric soil samples, we adopted the standardized DNeasy PowerLyzer Kit protocol (Qiagen, Hilden, Germany). Heterogeneous DNA was isolated from three independent soil samples (each weighing 0.350 g). After that, the DNA isolates were compared to select DNA with good yield and purity. The quality and usefulness of the DNA obtained was verified by PCR reactions, using primers [30] 27F (5′-AGAGTTTGATCATGGCTCAG-3′) and 518R (5′-GTATTACCGCGGCTGCTGG-3′) in the following PCR conditions: 98 °C for 5 min, 30 cycles of 98 °C for 35 s, 54 °C for 45 s, 72 °C for 60 s, and 72 °C for 5 min. 5× FIREPol^®^ Master Mix (Solis BioDyne, Tartu, Estonia) was used for the PCR reaction. After receiving positive results of the PCR reaction, triplicate samples of soil DNA were pooled as recommended by Kuźniar et al. [25].

### 2.3. Next-Generation Sequencing

Metabarcoding was performed based on the hypervariable V3–V4 region of the 16S rRNA gene [31]. In this approach, a 341F and 785R primer set was applied for both the amplification of the selected region and the preparation of the library [31]. The PCR reaction was carried out using Q5 Hot Start High-Fidelity 2X Master Mix (New England Biolabs Inc., Ipswich, MA, USA) as described by Wolińska et al. [29].

Next Generation Sequencing (NGS) was performed by Genomed S.A. (Warsaw, Poland) on a MiSeq sequencer (Illumina, San Diego, CA, USA) in paired-end (PE) technology, 2 × 300 nt, using an Illumina v2 kit (San Diego, CA, USA). The preliminary analysis of the data obtained was carried out with MiSeq Reporter (MSR) v2.6 software (Illumina, San Diego, CA, USA).

### 2.4. Bioinformatic Analyses

The identified sequences are available under accession number PRJNA622671 (GenBank Database, NCBI: https://www.ncbi.nlm.nih.gov/bioproject/PRJNA622671, accessed on 2 April 2020).

The analyses were conducted using Qiime2 versions 2019.7.0, 2021.4.0, DADA2 plug-in and in R version 4.1.1. Adapter sequences were removed from all reads using Trim Galore. Based on the sequence quality plots, the forward and reverse reads were trimmed to 280 and 240 bp, respectively, and the primer sequences were removed from all reads. The filtering parameters were set to the default values. PERMANOVA and alpha- and beta-diversity analyses were carried out using Qiime2. The taxonomy of each operational taxonomic unit (OTU) sequence was analyzed by the RDP Classifier (the latest modified version 18 available here: https://zenodo.org/record/4310151#.Yk1ZhuhBxPY, accessed on 12 April 2021) against the 16S rRNA database. We attached the taxonomy_OTU file (Table S1). The resulting taxonomy and read-count tables constructed in DADA2 plug-in were successfully imported into the phyloseq package version 1.36.0 in R. The top 10 core microbiomes according to the membership-based method were selected as the most numerous OTUs shared between all 4 wheat and all 4 legume samples respectively. Since microbiome data represents relative abundances, it is compositional in nature. The standard deviation of the relative abundance of OTUs was further calculated and compared for composition-based methods. The core microbiome was analyzed with the usage of R microbiome package version 1.14.0. The functional characteristics of the microbiome were predicted using the FAPROTAX database (http://www.loucalab.com/archive/FAPROTAX/lib/php/index.php?section=Download, accessed on 12 April 2021). The definition method of membership is an analysis of shared taxa within two or more microbiomes; these taxa are represented by overlapping parts in a Venn diagram. In this study of the rhizosphere, OTUs that were shared in the 4 (W) and 4 (L) samples were defined as the core microbiome [17]. While, the definition method of composition is analysis based on the principle of coexistence, the weighting of OTUs is considered, that is, shared OTUs with similar proportions are defined as the core microbiome. In accordance with the definition in our studies, the core microbiome is based on the results of membership analysis, the standard deviation of the relative abundance. OTUs with an SDRA threshold lower than 0.01 are defined as the core microbiome [17]. According to Sansupa and co-workers [32], FAPROTAX can be used for a fast-functional screening or grouping of 16S derived bacterial data from terrestrial ecosystems. What is more, these authors also tested this interactive tool for microorganisms among other agricultural-rhizosphere soil. The functional assignments depend heavily on taxonomic information at the genus, species, or strain level of a given taxon of bacteria and archaea in the FAPROTAX database. The statistical analyses were performed using STATISTICA 12.0 software (Hamburg, Germany).

A total of 4204 (W1); 5028 (W2); 5006 (W3); and 4800 (W4) bacterial sequences, 3107 (W1); 4427 (W2); 3874 (W3); and 3907 (W4) bacterial OTUs, and phyla from 7 bacteria (W1)–9 (W3) were obtained, respectively, after NGS in this study. Rarefaction curves (Figure S1) for these samples showing the diversity detected (OTUs) were compared with the predicted total reads. The *x* axis represents the number of sequences sampled while the *y* axis represents the measures of the OTUs detected. The legend on the bottom shows the correspondence between the curves and the samples.

## 3. Results

### 3.1. Top 10—Wheat Rhizospheric Core Microbiome Defined with the Membership-Based Method

**Figure 1:**
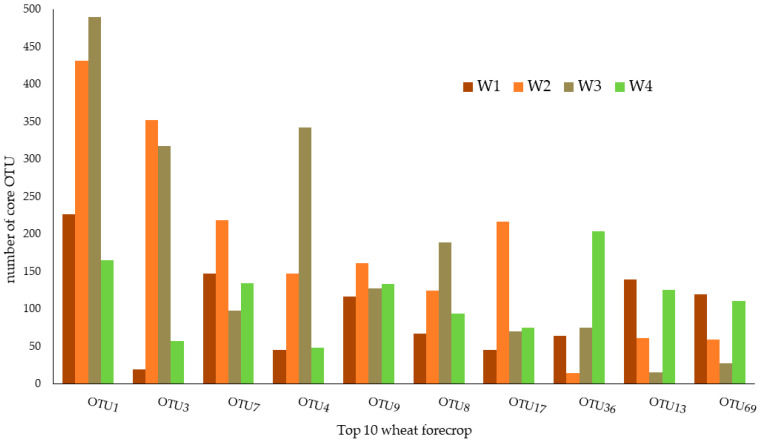
Taxonomic composition of the core bacterial microbiome of rhizosphere soil subjected to the wheat forecrop (W), which repeats from 1 to 4, respectively.

Figure 1 and Table 1 represent ten core OTUs that identify the core microbiomes of rhizospheric soil subjected to the wheat forecrop (W) defined with the membership-based method. The relative abundance of the core microbiome accounted for 0.1976% of the total sample (Table 1). Among the ten OTUs, the dominant taxon was OTU1, with a relative abundance of 0.334%. In contrast, the lowest relative abundance was found in the case of OTU12 (0.0118%).

These OTUs belong to three phyla (Actinobacteria, Nitrospirae, Proteobacteria) and were classified into five classes (Actinobacteria, Alphaproteobacteria, Betaproteobacteria, Nitrospira, Thermoleophilia) and six orders (Burkholderiales, Nitrospirales, Micrococcales, Rhizobiales, Sphingomonadales, Solirubrobacterales). Descending to a lower taxonomic level, the OTUs determined with this method represented six families: Baekduiaceae, Bradyrhizobiaceae, Hyphomicrobiaceae, Nitrospiraceae, Micrococcaceae, and Sphingomonadaceae and five genera: *Baekduia*, *Bradyrhizobium*, *Nitrospira*, *Pseudarthrobacter*, and *Sphingomonas* (Figure 1; Table 1).

**Figure 2:**
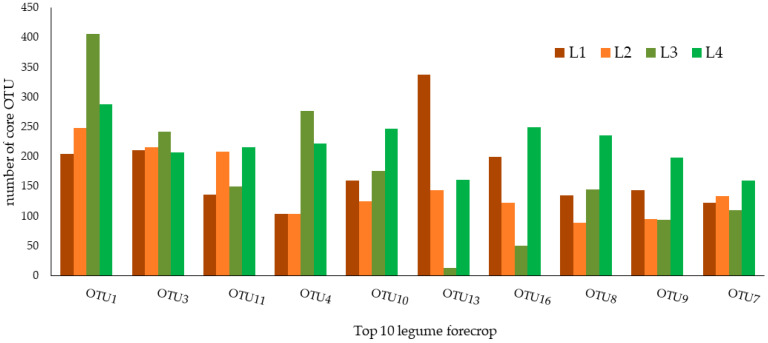
Taxonomic composition of the core bacterial microbiome of rhizosphere soil subjected to the legume forecrop (L), with repeats from 1 to 4, respectively.

The core microbiome of the wheat rhizospheric soil subjected to the legume forecrop (L) are presented in Figure 2 and Table 2. In general, the relative abundance of the core microbiome of soil subjected to the L forecrop remained at a similar level to that in the W forecrop variant (Figure 1; Table 1) and accounted for 0.2006%. Among these top ten OTUs, OTU1 seemed to be the dominant core taxon (0.0325%), whereas OTU7 was the taxon with the lowest relative abundance in this treatment (0.0149%).

The identified OTUs belong to three phyla: Actinobacteria, Nitrospirae, and Proteobacteria; three classes: Actinobacteria, Alphaproteobacteria, and Nitrospira; and the following orders: Burkholderiales, Nitrospirales, Micrococcales, Rhizobiales, Gp3, and Gp16. These OTUs were also classified into four families: Bradyrhizobiaceae, Hyphomicrobiaceae, Nitrospiraceae, and Micrococcaceae and two genera: *Nitrospira* and *Pseudarthrobacter* (Figure 2; Table 2).

### 3.2. Core Microbiome Defined with the Composition-Based Method

With the use of the composition-based method, we determined the core microbiome of rhizospheric soils subjected to the W forecrop management. In this analysis, the threshold (<0.01) was used; thus, five amplicon sequence variants (ASV) were defined as the core microbiome, the members of which belonged to different taxa. It should be emphasized that most of the identified members of the core microbiome represented the most abundant taxa in the studied soils. These ASV belonged to two phyla: Actinobacteria and Proteobacteria (Table 3); three classes: Thermoleophilia, Alphaproteobacteria, and Betaproteobacteria; and four orders: Sphingomonadales, Rhizobiales, Solirubrobacterales, and Burkholderiales. Additionally, three families: Hyphomicrobiaceae, Bradyrhizobiaceae, and Sphingomonadaceae and two genera: *Sphingomonas* and *Bradyrhizobium* were identified (Table 3). The relative abundance of the core microbiome accounted for 6.425% of the total sample. Among the five core ASVs, the dominant core taxon was represented by species belonging to *Sphingomonas* sp., with a relative abundance of 1.638%.

In the rhizosphere soil subjected to the L forecrop, the following ASV representing two phyla: Nitrospirae and Proteobacteria (Table 4) and three classes: Nitrospira, Alphaproteobacteria, and Betaproteobacteria were determined. Additionally, three orders: Burkholderiales, Rhizobiales, and *Rhodoplanes* sp.; the family Hyphomicrobiaceae; and the genus *Nitrospira* were noted. The relative abundance of the core microbiome accounted for 4.253% of the total sample. Among the five core ASVs, the dominant core taxon was represented by Burkholderiales, with a relative abundance of 1.240% (Table 4).

### 3.3. Core Microbiome Identified Based on Functional Redundancy

Some of the detected microbial community members in the L and W forecrop variants remained unclassified and represented uncultured species, whereas the FAPROTAX database relies on characterized strains. Finally, only identified microorganisms were selected for the functional core analysis.

**Figure 3:**
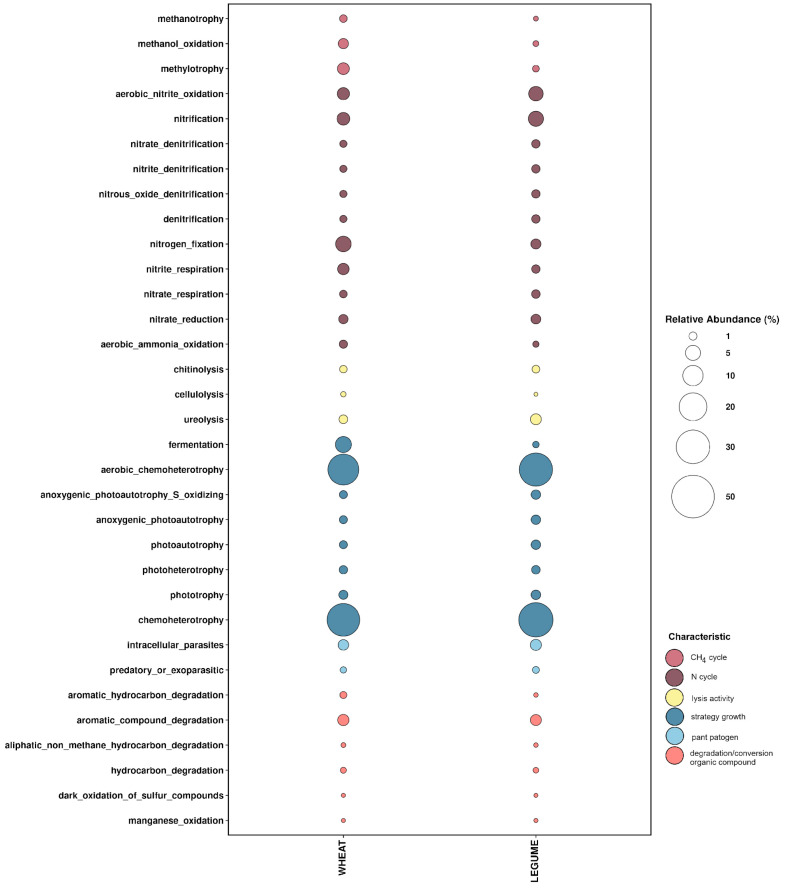
Relative abundance of different functions (*y* axis) based on the FAPROTAX database in the wheat and L forecrop samples. The size of the balloon indicates the relative taxonomy-based functional capacities of the microbial community.

The functional FAPROTAX predictions indicated that chemoheterotrophy was the major driving force in soil metabolism in both W and L forecrop variants (Figure 3). At a similar level, the same OTUs appeared also in the aerobic chemoheterotrophy category in the soil in both forecrop variants. Interestingly, the analysis of the predicted growth strategies of the microorganisms inhabiting the rhizosphere revealed that the fermentation activity of bacteria from the rhizosphere of the soil in the W forecrop variant was definitely more likely. In that case, we found a significant difference between the percentages of the function assignment in the FAPROTAX interactive tool to the total detected OTUs, as the functional assignment in the W forecrop rhizosphere soil variant accounted for 5.806% (*p* = 0.005), in contrast to the soil samples from the L forecrop variant, where it was 0.578%.

Lower intensity of nitrification and aerobic nitrate fixation was confirmed, and the levels of the processes were slightly higher in the L than W forecrop variant; however, nitrogen fixation was more intensive in the W forecrop variant (Figure 3). In total, 5.435% and 1.837% of OTUs detected in the wheat rhizosphere soil from the W and L forecrop variants, respectively, were functionally assigned to nitrogen fixation by FAPROTAX.

A similar level of the degradation of aromatic compounds was found in both studied variants (W-2.452%; L-2.304% of all OTUs), whereas the processes of methylotrophy and methanol oxidation were more intensive in the W forecrop variant. In this case, we found a significant disproportion between the functional assignment of the W forecrop rhizosphere soil (W_2.805%) and the L forecrop (L_0.587%) (*p* = 0.0048).

**Figure 4:**
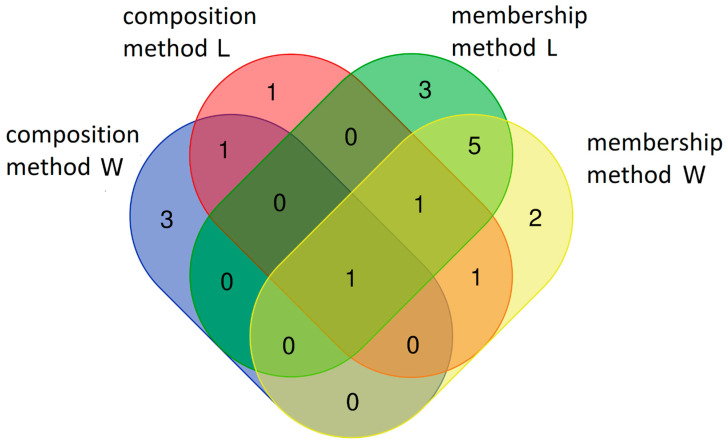
Differences and similarities in the core microbiome of rhizospheric soil during the growth of the selected wheat cultivars planted with different forecrops.

The functional lytic activity (mainly chitinolysis and cellulolysis) of the bacteria colonizing the rhizosphere was similar in the two forecrop variants used, with the exception of ureolysis activity (Figure 4). In the rhizospheric soil, we functionally assigned lytic functions of 1.089% and 0.892% of OTUs in the W and L forecrop variants, respectively. The situation is different with regard to microbial ureolysis, as the core microbiome activity was nearly twice as high in the wheat rhizospheric soil after the L forecropping (2.258% of all OTUs) than in the wheat rhizospheric soil after the W forecropping (1.250%). Noteworthy is the similar functional trend in the plant pathogen activity referred to as intracellular_parasite and predatory or exoparasitic assigned in both ecological niches. We assigned 2.583% and 3.018% of the total detected OTUs in the W and L forecropping variants, respectively, in the FAPROTAX database. The analysis of the rhizosphere core microbiome identified based on functional redundancy indicated the variation in bacterial community function within studied wheat varieties after different forecrops.

### 3.4. Summary of the Core Microbiome Composition Determined with the Different Methods

The results of the two methods used to define the core microbiome of the rhizospheric soils exhibit both similarities and differences, as illustrated in Figure 4. One difference is the number of taxonomic units composed by the core microbiome determined with the membership method (ten OTUs) and the number of units determined with the composition and connectivity methods (five OTUs each).

In the rhizospheric soil subjected to the L forecrop, the members of the core microbiome identified with the composition-based method overlapped with those determined with the membership-based method. OTU11 was the only overlapping taxon (*Rhodoplanes* sp.).

No common taxa were recorded with the two methods in the microbiome originating from the rhizosphere soil with the W forecrop. The membership-based method revealed five overlapping core taxa in the microbial cores of the rhizosphere soil with different forecrops, i.e., OTU8 (*Pseudarthrobacter* sp.), OTU4 (*Pseudarthrobacter* sp.), OTU13 species assigned to the order Gp3, OTU7 Rhizobiales sp., and OTU9 (*Bradyrhizobium* sp.).

In addition, three unique rhizosphere soil microbial core taxa were identified in the W forecrop variant with the composition-based method: OTU151 (Solirubrobacterales), OTU883 (Burkholderiaceae), and OTU2 (*Sphingomonas* sp.). The composition method revealed one unique soil core taxon, i.e., OTU6 (Rhizobiales), in the L forecrop variant.

## 4. Discussion

Mohanram and Kumar [30] suggested the concept of a biased rhizosphere, which is based on the interactions among various components of the rhizosphere, e.g., plant roots and soil microbiome, and is intended to elucidate the complex rhizospheric intercommunications. It is known that rhizobacterial populations are recruited primarily from the bulk soil, but they are preselected by, e.g., an excess of released root carbon; hence, bacterial diversity is generally lower and bacterial networks are less stable in the rhizosphere [30].

Here, we compared the diverse core microbiome attributes of wheat rhizosphere communities in L and W forecrop variants determined with the use of three different methods. The current study analyzed the core microbiome in eight rhizospheric soil samples based on three methods (membership, composition, and functionality of the core microbiome) commonly used in the literature. The impact of location, land use history, cultivar, and crop species is widely discussed in the literature on the rhizomicrobiome [32,33,34]. The present analyses aimed at systematic evaluation of the impact of different determination methods on the wheat core rhizomicrobiome to provide new knowledge in this scientific area.

In the first method, the determination of the core rhizomicrobiome was based on identification of abundant taxa occurring in rhizospheric soil, which were highly competitive with other microorganisms or were vertically transmitted, recruited, selected, and inherited through evolution [17]. This membership-based method is commonly referred to as Top 10, because the ten abundant taxa widely present in the soil samples were identified as members of the core rhizomicrobiome in this study.

Most of these members belong to three phyla: Proteobacteria, Actinobacteria, and Nitrospirae in both forecrop variants. Of note, Proteobacteria and Actinobacteria are in general classified as dominant taxa in the soil environment [24], whereas Nitrospira representatives are classified as either dominants or subdominants in soils, with a crucial role in both ammonia- and nitrite-oxidation processes [35,36]. Simonin and co-workers [19] observed that 177 taxa (2 archaea, 103 bacteria, 41 fungi, and 31 protists) were consistently detected in the wheat rhizosphere, constituting a core microbiome. These authors revealed that these core taxa were highly abundant, which is in agreement with our findings, where these taxa represented 50% of the reads. Our results indicated that the relative abundance of the core taxa varied greatly across all samples (min = 0.0118%, max = 0.334%). The relative abundance of the core rhizomicrobiome of soil accounted from 0.2006 to 0.0340%. In turn, Simonin and co-workers [19] reported that the relative abundance of the core taxa varied greatly across wheat rhizosphere soils from four different countries (Cameroon, France, Italy, Senegal) (min = 0.02%, max = 5.7%). Furthermore, these authors showed that *Bradyrhizobium japonicum* (72% of samples) and *Arthrobacter* sp. (70%) were the most prevalent bacterial taxa.

Hamonts et al. [37] have proven that the drivers type of plant, growing region, sugarcane variety, crop age, and plant disease attack all explained significant fractions of the variation observed in sugarcane-associated bacterial and fungal community assemblages. Other results obtained by Zhalnina et al. [38] suggested that the combination of these plant exudation traits and microbial substrate uptake traits contributes to a metabolic synchronization that underlies microbial community assembly patterns observed in the rhizosphere. Considering the above discussion, therefore, we study core compared with three different methods. The conclusions of the three methods of microbiome core definition suggest the presence of generalists, i.e., some representative microorganisms from Proteobacteria, Actinobacteria, Hypomicrobiaceae, Bradyrhizobiaceae, *Sphingomonas* sp., in the wheat rhizomicrobiome. In support of these results, it should be mentioned that Jacquiod et al. [39] found a dominance of Proteobacteria, Actinobacteria, and Bacteroidetes OTUs in wheat core. These authors reported that plant genotype and phenotypic plasticity had the most influence on the rhizosphere microbiota, whereas inputs had only marginal effects [39]. Most of the Hyphomicrobiaceae were oligotrophic species, and the species of Sphingomonadaceae are able to utilize a wide diversity of organic compounds and to grow and survive under low-nutrient conditions [40]. The keystone species Solibacteraceae of the *S. chinensis* forests were also demonstrated to be active in carbohydrate mineralization [41].

*Nitrospira* sp. were widely detected in rhizosphere soils [42]. Our studies indicated that *Nitrospira* sp. contribute to the core. The mentioned strain can perform both ammonia and nitrite oxidation to produce nitrate [43]. The currently available knowledge suggests that the preferred niches of *Nitrospira* sp. may not be restricted in low nutrient environments, especially in soils. Studies by Li et al. [34] of terrestrial ecosystems suggest that *Nitrospira* are not strictly oligotrophic, rather both oligotrophic and copiotrophic, with a broader ecological niche breadth. This may explain its presence in the core microbiome of the rhizosphere [44].

In our study, we also used the composition-based method to identify the core microbiome. Dong et al. [17] found that this method emphasizes the abundance of OTUs in each sample. We defined five ASVs as the core microbiome. This method revealed that the relative abundance of the core rhizomicrobiome accounted from 4.253 to 6.425% of the total sample. Among the five core ASVs, the dominant core taxon was represented by *Sphingomonas* species, with a relative abundance of 1.638% (W forecrop) and an undetermined taxon from the order Burkholderiales, with a relative abundance of 1.240% (L forecrop). We used the composition-based method to highlight the significantly greater relative abundance of core rhizomicrobiome. Dong et al. [17] reported that this method showed the relative abundance of the most core microbiome. Consequently, the composition-based method presented taxa that were neither dominant nor rare and consistently represented the intermediate taxa group. Our results indicated that the core rhizomicrobiome identified by the composition-based method belonged to two phyla: Nitrospira and Proteobacteria (L forecrop) as well as Proteobacteria and Actinobacteria (W forecrop). Jochum et al. [45] indicated that Proteobacteria and Bacteroidetes were the most abundant phyla in the analyzed wheat rhizomicrobiome, followed by Actinobacteria and Firmicutes.

In the analysis of the core rhizomicrobiome, it is also important to highlight the physiological functions of microorganisms that make up the basic core. There are reports in the literature that highly abundant taxa may not always have an important function in a given ecological niche. Dong et al. [17] have shown that not all taxa of microorganisms classified as the core microbiome of *Eucommia ulmoides* bark have a significant effect on the physiology of the plant host. A similar situation may exist in the soil environment, given its complexity. For example, *Bradyrhizobium* sp. belongs to microorganisms with lower abundance; nevertheless, we are well aware that *Bradyrhizobium* species are biologically important in soils, as they serve a wide range of biochemical functions, including photo-synthesis, nitrogen fixation during symbioses, denitrification, and degradation of aromatic compounds [46,47]. Therefore, we analyzed the core rhizomicrobiome using the FAPROTAX database in line with the results obtained by Sansupa et al. [32], who evidenced that the source of the sample is not a primary factor limiting the application of FAPROTAX, and this tool can be successfully applied to soil samples. This analysis showed chemoheterotrophy followed by aerobic chemoheterotrophy as predominant functions in all samples (Figure 3). The occurrence of chemoheterotrophic microorganisms with an aerobic mode of respiration is not surprising due to the release of oxygen around the roots [48]. However, when we focused on more specific functions, the result showed differences in the dominant functions involved in biogeochemical cycling of carbon derived from rhizospheric soil. Ling et al. [35] reported that genes involved in organic compound conversion, nitrogen fixation, and denitrification were strongly enriched in the rhizosphere (11–182%), while genes involved in nitrification were strongly depleted. Importantly, this functional prediction was done based on data from the database. In contrast, our results revealed a similar level of assigned functions of denitrification, nitrification, and nitrogen fixation (Figure 3). The differences in these processes are probably related to the fact that the substrate for denitrification, i.e., ammonium ions, may not accumulate in the rhizosphere of agricultural soils. Our functional analysis showed differences in the physiological profile of the rhizomicrobiome. The issue of metabolic footprints related to physiological differences may be worth highlighting. The first example is the diversification in the fermentation level. Metabolic footprints provide metrics for the magnitudes of ecosystem functions and services provided by component organisms of the soil food web [49,50]. There are studies consisting of metabolic footprint analyses of metabolites that discriminate single and mixed yeast cultures at two key time-points during mixed culture alcoholic fermentations [51]. As a result of the comparison of the two rhizospheres in the different forecrop variants, we proposed that there is a potential impact on the phytopathic activity rhizospheric microorganisms, which may be regarded as a metabolic trace from root exudates and plant residues from the forecrops used. A similar phenomenon of metabolic memory is characteristic for nematodes. It conveys information about the composition of nematodes (bacterivores, omnivores, fungivores, herbivores, and predators) and their impact on the soil food web structure.

We analyzed the core rhizomicrobiome as all sequence reads from the rhizospheric soil (wheat cultivar). Using three methods, we identified the core microbiome as specific to rhizospheric soil after forecropping. Initially, we assumed the presence of differences resulting from the use of the different wheat varieties; however, the analysis of the data showed that the differences were insignificant. Ling et al. [35] also emphasized that even when genotypic and environmental differences were taken into account, certain similarities in the selection of microorganisms common to the rhizosphere were still observed. In spite of the genetic and environmental changes that may occur in this ecological niche, there are certain bacteria (as shown by the taxonomic analysis) that constitute the core microbiome (some representative microorganisms from Proteobacteria, Actinobacteria, Hyphomicrobiaceae, Bradyrhizobiaceae, and *Sphingomonas* sp.). We propose that the identified populations of microorganisms forming the generalist core rhizomicrobiome are widespread across multiple plant microhabitats and determine the ecological role of core species in microbial interaction networks. However, there is a group of specialists represented by a small community, which is strongly connected with other microbes or is essential for maintenance of the sustainability of certain metabolic pathways.

The values of OTU relative abundance presented in the data are relatively low (not all of them). There is such an ecological concept that includes core taxa that have the highest abundance or occupancy, simply because there are more robust patterns apparent of the taxa that are observed more frequently. However, recent works suggest that taxa that conditionally contribute to the microbiome may not meet the inclusion criteria for ‘core’ based on their abundance-occupancy, but then it is suggested using complementary approaches to identify these responsive taxa [42,43]. Our results are correlated with the concept demonstrated by Shade and Gilbert [43], who indicated that taxa that are typically in low abundance but occasionally achieve prevalence were shown to contribute to patterns of microbial diversity. What is more, interpretations of ecological biodiversity dynamics are essential to understand community stability of the rhizosphere.

Therefore, we recommend that, in rhizospheric soil subjected to the L forecrop, the OTU11 taxa (*Rhodoplanes* sp.) may be included in the group of specialists. Furthermore, regardless of the method used to determine the core, these taxa were part of the core rhizomicrobiome. The literature data indicate that *Rhodoplanes* species support the soil ecological food web by being primary producers [5].

## 5. Conclusions

In summary, we conclude that attention should be paid to the methods of identification of the core rhizomicrobiome with the assumption that a single method cannot indicate the core microbiome, especially in niches with high microbial richness dynamics (e.g., the soil environment). Combining multiple methods for determination of the core microbiome can help pinpoint the underlying microbiome at different levels and thus provide a better understanding of ecological processes. What is more, interpretations of ecological biodiversity dynamics are essential to understand the stability of the rhizospheric community. The abundance of taxon data in the core is not the main ecological factor, because just low abundance, occasionally achieving prevalence, was shown to contribute to ecological patterns of microbial diversity. Therefore, the identification of the core rhizomicrobiome is particularly important for understanding the general principles for the selection of microorganisms around living roots and harnessing the power of the microbiome for agricultural practice.

## Figures and Tables

**Table 1:** Relative abundance of the core bacterial microbiome of rhizosphere soil subjected to the wheat forecrop (W).

OTU ID	Taxonomy	Relative Abundance
OTU1	Hyphomicrobiaceae	0.0334
OTU2	*Sphingomonas* sp.	0.0328
OTU14	Bradyrhizobiaceae	0.0213
OTU5	Burkholderiales	0.0207
OTU3	*Nitrospira* sp.	0.0199
OTU7	Rhizobiales	0.0153
OTU26	*Baekduia* sp.	0.0145
OTU4	*Pseudarthrobacter* sp.	0.0144
OTU9	*Bradyrhizobium* sp.	0.0135
OTU12	*Bradyrhizobium* sp.	0.0118

**Table 2:** Relative abundance of the core bacterial microbiome of rhizosphere soil subjected to the legume forecrop (L).

OTU ID	Taxonomy	Relative Abundance
OTU1	Hyphomicrobiaceae	0.0325
OTU3	*Nitrospira* sp.	0.0248
OTU11	Bradyrhizobiaceae	0.0206
OTU4	*Pseudarthrobacter* sp.	0.0197
OTU10	Gp16	0.0195
OTU6	Rhizobiales	0.0182
OTU13	Gp3	0.0173
OTU16	Bradyrhizobiaceae	0.0169
OTU8	*Pseudarthrobacter* sp.	0.0165
OTU7	Rhizobiales	0.0149

**Table 3:** Taxonomic composition of the core bacterial microbiome of rhizospheric soil subjected to the W forecrop determined with the composition-based method. ASVs with an SDRA threshold lower than 0.01 were defined as the core microbiome.

OTU ID	Wheat Forecrop	
	Taxonomy	Relative Abundance (%)
OTU1	Hyphomicrobiaceae	1.232 ± 0.1780
OTU3	*Nitrospira* sp.	0.913 ± 0.2790
OTU7	Bradyrhizobiaceae	0.3840 ± 0.0033
OTU4	*Pseudarthrobacter* sp.	0.6180 ± 0.0021
OTU9	*Bradyrhizobium* sp.	0.3380 ± 0.0016
OTU8	*Pseudarthrobacter* sp.	0.2950 ± 0.0003
OTU17	*Nitrospira* sp.	0.1350 ± 0.0005
OTU36	*Streptosporangium* sp.	0.3041 ± 0.0023
OTU13	Acidobacteria_Gp3	0.2954 ± 0.0001
OTU69	*Devosia* sp.	0.2575 ± 0.0001

**Table 4:** Taxonomic composition of the core bacterial microbiome of rhizospheric soil subjected to the L forecrop.

OTU ID	Legume Forecrop	
	Taxonomy	Relative Abundance (%)
OTU1	Hyphomicrobiaceae	1.0240 ± 0.1810
OTU3	*Nitrospira* sp.	0.7360 ± 0.0930
OTU11	*Rhodoplanes* sp.	0.6750 ± 0.1420
OTU4	*Pseudarthrobacter* sp.	0.5760 ± 0.2663
OTU10	Acidobacteria_Gp16	0.5599 ± 0.1328
OTU13	Acidobacteria_Gp3	0.6360 ± 0.1460
OTU16	Rhizobiales	0.4457 ± 0.0129
OTU8	*Pseudarthrobacter* sp.	0.3634 ± 0.0500
OTU9	*Nitrospira* sp.	0.3072 ± 0.0663
OTU7	Bradyrhizobiaceae	0.3451 ± 0.1095

## Data Availability

High throughput sequencing data for this paper are available from the NCBI under accession number PRJNA622671.

## Supplementary Materials

Supporting information can be downloaded at: https://www.mdpi.com/article/10.3390/biology12081067/s1, Figure S1. Rarefaction curves for soil samples studied and Taxonomy_OTU. Table S1: The taxonomy_OTU file.
