# Supplementary figures and images for: Ecological Diversity of Bacterial Rhizomicrobiome Core during the Growth of Selected Wheat Cultivars
Source: Biology (Basel). 2023 Jul 30;12(8):1067. doi: 10.3390/biology12081067 (PMC10451756; doi:10.3390/biology12081067)

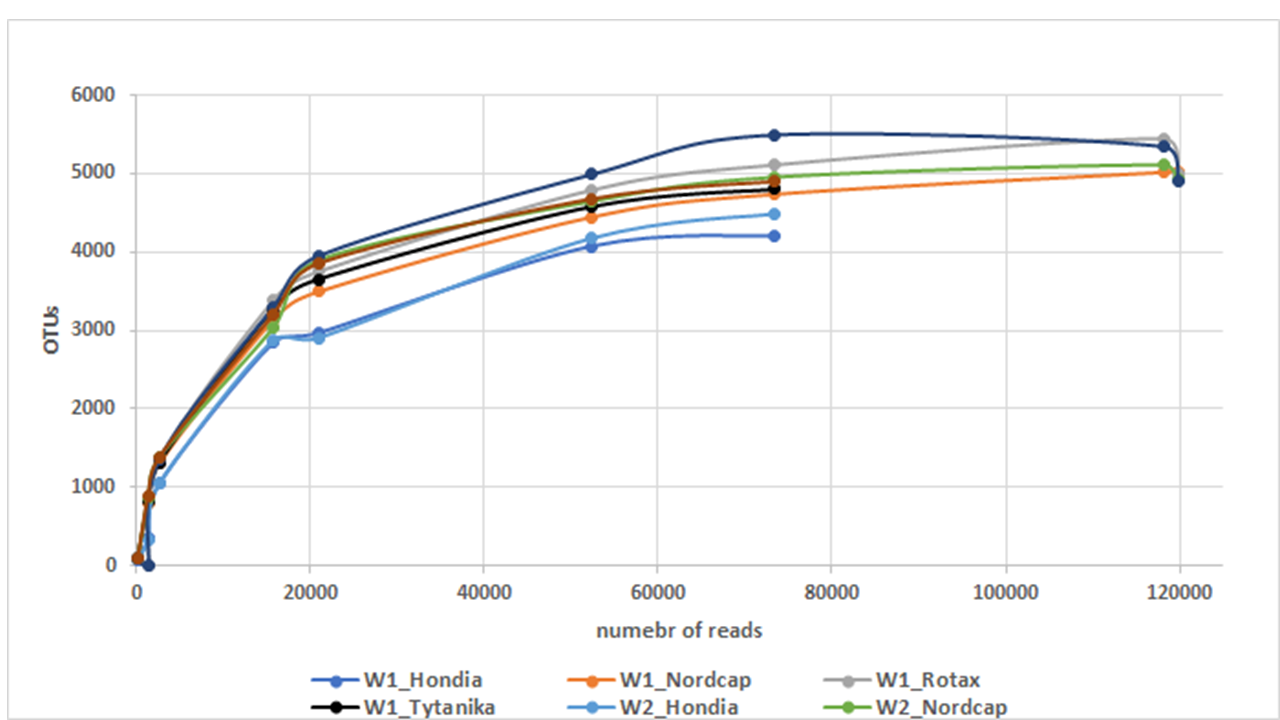

Supplement: Supplementary file 1 [file biology-12-01067-s001.zip › biology-2504294-supplementary.tif]
